# Let this be a safe place: a qualitative study into midwifery care for forcibly displaced women in the Netherlands

**DOI:** 10.1186/s12913-024-11852-w

**Published:** 2024-11-29

**Authors:** J. B. Tankink, A. E. H. Verschuuren, J. P. de Graaf, E. I. Feijen-de Jong, P. J. A. van der Lans, M. E. T. C. van den Muijsenbergh, A. Franx, B. Goodarzi

**Affiliations:** 1 Department of Obstetrics & Gynecology, Erasmus Medical Center, Rotterdam, the Netherlands; 2 Department of Health Sciences, Global Health Unit, University of Groningen, University Medical Center Groningen, Groningen, the Netherlands; 3 Midwifery Academy Amsterdam Groningen, InHolland, Netherlands & Amsterdam UMC location Vrije Universiteit Amsterdam, Midwifery Sciences, Amsterdam, the Netherlands; 4 Public Health Services (GGD), Amsterdam, the Netherlands; 5 Department of Primary and Community Care, Radboud University Medical Center, Nijmegen, the Netherlands

**Keywords:** Midwifery care, Asylum Seekers, Refugees, Barriers, Equitable care, Pregnancy and chidbirth, Qualitative, Netherlands

## Abstract

**Background:**

Forcibly displaced women in the Netherlands face increased chances of perinatal mortality and other adverse pregnancy and childbirth outcomes compared to the resident country population, which has been linked to suboptimal care. This study was conducted to gain insights from the experiences of Dutch midwives to inform and enhance the provision of tailored and equitable care for forcibly displaced women.

**Methods:**

We conducted a qualitative study using semistructured interviews with community midwives who provide care for forcibly displaced women (asylum seekers and recognized refugees) in the Netherlands. Through thematic analysis, we identified the barriers midwives encounter in providing care and explored their strategies for navigating these barriers, aiming to inform recommendations that advance equitable care provision.

**Results:**

Interviews with eleven midwives revealed barriers across three thematic levels: (1) the interactional level, where barriers related to language and interpreters, cultural differences, and building trust impeded positive interactions between midwives and forcibly displaced women; (2) the organizational level, where barriers concerning relocations of asylum seekers, delays in accessing care, and interdisciplinary collaboration impeded optimal care; and (3) the contextual level, where barriers related to women’s housing conditions, the resettlement process and the mental health of forcibly displaced women impeded midwives’ to respond to clients’ needs. These levels of barriers culminated in a core theme of imbalance between midwives’ expanded responsibilities and the limited resources and strategies available to them in care for forcibly displaced women. This imbalance forced midwives into multiple roles, increased both the practical and emotional burden on them, and undermined their ability to provide optimal, equitable care.

**Conclusions:**

To enhance the provision of equitable pregnancy and childbirth care for forcibly displaced women in the Netherlands, it is crucial to target the imbalance between the responsibilities that midwives bear and the resources available to them. This requires dismantling barriers at the interactional, organizational and contextual level of care through targeted policy interventions. Structural determinants that perpetuate the imbalance in midwives’ work and restrict their scope of influence, such as restrictive migration policies that contribute to socioeconomic marginalization and poor housing conditions, need to be addressed. Ultimately, midwives themselves require more support and education to recognize and combat injustices in pregnancy and childbirth care for forcibly displaced women.

**Supplementary Information:**

The online version contains supplementary material available at 10.1186/s12913-024-11852-w.

## Introduction

An increasing number of people of reproductive age are forcibly displaced from their home country due to threats to their lives or livelihood, seeking safer residence elsewhere [1]. In many of their destination countries, however, forcibly displaced people face reduced chances of a positive outcome of pregnancy and childbirth compared to local populations [2–4]. In the Netherlands, recent studies showed a two- to sevenfold increased risk of perinatal mortality in women* with a forced migration background compared to resident women [5, 6]. These findings add to a body of evidence demonstrating maternal and perinatal health inequities in the context of forced migration [7, 8]. 

Suboptimal healthcare significantly contributes to increased rates of adverse outcomes of pregnancy and childbirth in forcibly displaced populations. Several studies demonstrated specific suboptimal care factors, such as unresolved language barriers, inadequate responses to complaints, and culturally insensitive care [9, 10]. A recent Dutch study confirmed that suboptimal care factors delay care seeking and access and receipt of quality care among forcibly displaced women, which ultimately may result in adverse outcomes [10, 11]. To prevent this, a national guideline outlines responsibilities and recommendations for pregnancy and childbirth care for asylum seekers in the Netherlands, although its scope and practical implementation remain limited [12, 13]. 

In the Netherlands, community care midwives provide comprehensive care throughout pregnancy and childbirth and often represent primary healthcare contacts for pregnant women who have recently undergone forced migration. A previous exploratory study revealed that Dutch midwives encounter a multitude of challenges, including issues related to communication with clients, continuity of care, and psychosocial care [13]. These findings indicate the need for a more detailed understanding of these challenges to unravel the mechanisms underlying suboptimal care and ultimately strengthen midwives’ position in combating health inequities.

The overarching aim of this study is to generate insights from midwives’ experiences that will inform and enhance the provision of tailored and equitable care for forcibly displaced women. Guided by two key research questions—what specific barriers do midwives face when caring for these women, and how do they manage these barriers to meet women’s needs?—this study aims to deepen our understanding of current care practices. The findings will facilitate critical reflection on these practices and guide the formulation of targeted recommendations to support midwives in their roles, and ultimately improve pregnancy and childbirth outcomes for forcibly displaced women.

* While we use the term “woman” in this article, it is intended inclusively to encompass all gender identities who give birth.

## Methods

### Design and study populations

This qualitative study was part of a larger research project on pregnancy and childbirth care for forcibly displaced populations in the Netherlands (the EGALITE project). We collected data through semistructured interviews with Dutch community care midwives who provide care to forcibly displaced women. Throughout the study, we use the term “forcibly displaced women” or the specific terms “asylum seekers” and “recognized refugees/refugees with a residence permit” to distinguish between women living in reception centers and women with a residence permit in regular housing. While acknowledging the limitations of these labels in capturing the diversity of experiences and identities within forced migration, these choices reflect the terminology most commonly used in clinical practice while distinguishing between different legal statuses in the Dutch setting.

### Setting

In the Netherlands, asylum seekers are accommodated in government-run reception centers managed by the Dutch Central Agency for the Reception of Asylum Seekers (abbreviated to the ‘COA’ in Dutch). These centers provide temporary housing and access to basic services such as food and healthcare while asylum requests are processed. The COA contracts different organizations to provide primary healthcare services in reception centers. During the period of this study, the main contracted provider was HealthCare Asylum Seekers (abbreviated to GZA in Dutch). Upon receiving a residence permit, recognized refugees are eligible for social housing and Dutch health insurance, thereby gaining access to the regular healthcare system.

Pregnant women living in asylum reception centers are referred by COA or GZA to a nearby community midwifery practice or hospital. In the Dutch pregnancy and childbirth care system, community midwives provide comprehensive care and consult medical specialists or refer women to hospitals when necessary. After birth, postpartum care assistants offer support in hospitals, women’s homes, or asylum reception centers, although they receive no specific training for this setting. During this study, governmental insurance covered the costs of professional telephone interpreter services for pregnancy and childbirth care for asylum seekers, but not for recognized refugees.

### Participant recruitment

We used purposive sampling to recruit midwives from nine midwifery practices that participated in the EGALITE project. These were regular community midwifery practices across different regions of the Netherlands, where a portion of the client population consisted of asylum seekers and recognized refugees. The midwives in this study were not exclusively specialized, but provided care to both the general population and forcibly displaced women. Besides geographical distribution, we approached midwives who varied in ages, years of experience, and cultural backgrounds to achieve a diverse sample. Midwives were invited to participate in the study via e-mail.

### Data collection

The first author (JT) conducted all the interviews in the presence of a student (SE). We used a semistructured topic guide for the interviews, which was developed through a literature review and field visits to three midwifery practices prior to the study. The field visits consisted of observations of antenatal consultations with forcibly displaced women and informal conversations with midwives. The first version of the topic guide included midwives’ general experiences with care and more specific questions regarding the barriers they encountered, their strategies to navigate these barriers, and the needs they indicated to improve tailored care to forcibly displaced women. As the interviews progressed from March to April 2021, emerging experiences and challenges were dynamically integrated into the topic guide. The most up-to-date version of the topic guide is included as supplementary material (Appendix 1).

The interviews ranged from 48 to 64 min, with one session including two midwives from the same practice by their request. One interview was conducted at the midwife’s workplace. Due to COVID-19, eight interviews were conducted via Microsoft Teams, and one interview took place outdoors. After completing interviews with eleven midwives, data saturation was reached.

### Data processing and analysis

The audio-recorded interviews were transcribed verbatim and were accessible only to the first two authors on a secured server of the Erasmus University Medical Center. We conducted a member check by inviting all midwives to review their transcripts for feedback or additional comments; five midwives agreed to receive their interview transcripts and confirmed the accuracy of the content.

We combined deductive and inductive approaches in thematic analysis, facilitated by ATLAS.ti software (version number 22.2.3) [14, 15]. Initially, two researchers (JT and AV) independently coded the first interview transcript. Codes were categorized into the domains of challenges identified in a previous survey study conducted among Dutch community midwives [13]. These domains included interdisciplinary collaboration, communication with clients, continuity of care, psychosocial support and addressing the needs of women in a vulnerable situation. As midwives shared barriers beyond the scope of these domains during the interviews, we proceeded with inductive coding and categorizing to identify new emerging themes. This iterative process of developing and adjusting themes was guided by reflective discussions within the research team to critically assess our analysis and interpretation [16]. 

No specific theoretical framework guided the analysis of this study. However, we applied an equity perspective as a lens for reflecting on and interpreting our results in the discussion section. Specifically, the concept of reproductive justice – which refers to the right to make autonomous decisions about parenthood in safe and supportive environments—was used to contextualize the barriers midwives encountered within the broader sociopolitical landscape of care.

### Positionality

The research team comprised PhD-level and senior researchers, midwives, a gynecologist and a general physician. All team members possess diverse academic, clinical and personal experiences in healthcare for forcibly displaced women. The first author and primary interviewer (JT) is a medical doctor and PhD candidate. Several shared social identity traits, such as gender, age and cultural background, likely facilitated rapport with the majority of midwives during the interviews. To mitigate biases linked to her “insider” status within the Dutch healthcare system, JT deliberately avoided making assumptions and actively invited participants to discuss practices and experiences they might consider self-evident. Nevertheless, the study’s focus on improving care for forcibly displaced women could have influenced midwives to provide socially desirable responses. While the topic guide was informed by the literature and field visits, we acknowledge that our team’s limited personal experience with forced migration might have narrowed our perspective and the breadth of data interpretation.

## Results

We approached nine midwifery practices, resulting in eleven individual midwives who agreed to participate in the study. Nine midwives were born in the Netherlands, while two midwives had a different country of birth, although none identified as refugees. The midwives’ ages ranged from 25 to 51 (M 34.7), with 2.5 to 17 years of experience with forcibly displaced populations (M 7.0).

Thematic analysis revealed that the barriers that midwives encountered in care manifested at three thematic levels: (1) the interactions between midwives and clients, (2) the organization of care, and (3) the context and living circumstances of forcibly displaced women. As a result of midwives’ experiences with these barriers, we identified an imbalance between increased responsibilities and limited resources in care for forcibly displaced women. The code tree is presented in Appendix 2.

### 1. Barriers at the level of midwife-client interactions

The first level of barriers affected the individual interactions between midwives and their forcibly displaced clients. These barriers were mostly related to language and interpreters in care, cultural differences, and building trust.

#### Language and interpreters

Almost all midwives described how language barriers affect the autonomy, access to information, and freedom of expression of refugee clients throughout pregnancy care, particularly during childbirth:

*“I notice how language plays an enormous role*,* because often people actually have an opinion*,* but well yeah—if you cannot express it*,* I’d also rather keep quiet. Otherwise*,* I might end up in a difficult situation where you keep asking questions that I can’t answer.”* (Midwife 5).

All midwives identified the absence of government-funded professional (telephone) interpreters for refugees with residence permits as a major barrier. In practice, midwives frequently relied on hand gestures or on the clients’ friends or relatives, including minors, to bridge language gaps, while interpreters were considered to be *“a luxury”*:

“*And I’m thinking about what you [the interviewer] just said*,* ‘don’t you find it difficult with an interpreter?’ But for us – I often talk to someone else*,* a sister or a friend or a child*,* so a [professional] interpreter can truly be a luxury*,* that what I am saying gets translated at least. Because with someone else – I say ten sentences and then they translate one thing*,* and then the lady says ‘yes’. Well*,* that was it (laughs). So I truly think the [professional] interpreter is a luxury*!” (Midwife 6).

Apart from financial barriers, midwives indicated that telephone interpreter services posed additional barriers because the required language was not always available, the quality of the interpreters varied, and the services were perceived as time-consuming. Even when interpreters were engaged in consultations, most midwives still reported barriers, such as miscommunication and difficulty conveying emotions.

To overcome communication barriers, several midwives felt that working with women-identifying interpreters often helped to make women with a background of forced migration feel more comfortable. They also mentioned the value of multilingual educational materials and the importance of reserving extra time for appointments.

#### Cultural differences

The majority of midwives described accommodating the diverse cultural backgrounds of their clients as a challenge in care provision. In particular, midwives noted that clients’ expectations and preferences regarding aspects such as the frequency of antenatal consultations or birthing methods often deviated from the norm in Dutch healthcare practices. In addition, a number of midwives observed that forcibly displaced women expressed their questions less openly than other clients. In addition to language barriers, midwives attributed this pattern to cultural differences, such as norms of respect for medical professionals. Furthermore, midwives often perceived forcibly displaced women as less demanding and more content with care. Several midwives mentioned being motivated by their clients’ gratitude.

Although only a few midwives mentioned having limited competence in handling cultural differences, most interviews contained examples of cultural stereotyping:

*“Usually*,* people from Syria are very motivated; they try to learn Dutch by themselves; they are well informed about everything*,* always on time. Well*,* from Eritrea*,* not everyone is like that*,* of course. There are also people who arrive on time and show up consistently*,* so not everyone – but there is a difference…”* (Midwife 8).

To manage cultural differences in care, midwives described having respectful dialogues regarding cultural values and habits as a way to connect with women. Several midwives emphasized that care for forcibly displaced women did not fundamentally differ from care for other women, who shared similar worries, wishes, and physiological aspects of pregnancy, *“which is conceived and progresses in the same way”* (Midwife 7).

#### Building trust

Midwives noted several barriers related to building a relationship of trust with forcibly displaced women. Besides language barriers and cultural differences, limited continuity of care was identified as a key barrier to trust. Continuity of care was most commonly hindered by relocations of asylum seekers or by transfer of pregnancy care from midwives to hospitals. Additionally, clients’ shame and fear, particularly regarding the immigration authorities, were mentioned as barriers.

To navigate these barriers, several midwives emphasized the value of becoming a familiar presence in locations near asylum reception centers. One midwife shared that having the same religious background as her clients was an asset with regard to building trust. Like other participating midwives, she highlighted the importance of patience and sensitivity to potential trauma when interacting with women:

*“And if someone truly does not want to share anything*,* then it is just done*,* then I always say*,* ‘I know you have been through something rough*,* I know it’s a process that can be painful but also beautiful. Know that I’m here for you*,* the moment you do want to talk about it. I’m here for you.’ And then – often that is just enough to open up a story.”* (Midwife 5).

### 2. Barriers in the organization of care

The second level of barriers concerned the organization of care for forcibly displaced women. These barriers were mostly related to the relocation of asylum seekers, delays in access to care, and interdisciplinary collaboration.

#### Relocations of asylum seekers

Midwives unanimously identified relocations between reception centers as a challenge that compromises the overall quality of care for asylum seekers. Almost all midwives experienced fragmented care due to the absence or delay of the transfer of medical information to subsequent care providers following relocations, which were often not communicated timely by the COA. In addition, relocations were considered physically burdensome and distressing for clients. They also diminished some midwives’ own sense of control:

*And as a result [of relocations]*,* you simply lose control from time to time. And that is where I struggle most*,* thinking ‘oh*,* so now you are gone and going somewhere else*,* is it going to be handled in the right way?’* (Midwife 1).

Midwives appreciated the national guideline for asylum seekers, particularly the recommendation against relocating women between the 34th week of pregnancy and the 6th week postpartum. However, multiple midwives had experienced relocations occurring outside this period without their consultation. To mitigate information loss during relocations, midwives tried to provide detailed handovers to the next care providers and, in some cases, provided printed pregnancy records to the women to carry with them.

#### Delays in access to care

Most midwives reported frequent delays in antenatal care initiation among forcibly displaced women, complicating risk assessment during pregnancy, especially when women arrived without any registered medical history. Missed antenatal appointments were also common, which midwives attributed to several barriers delaying care seeking and access. Besides relocations of asylum seekers, these included clients’ lack of transportation options and insufficient knowledge of the health system. Several midwives resorted to using their personal cars to transport clients from asylum reception centers to the hospital, due to unreliability of the taxi services that women are entitled to around childbirth. In addition, critical care was sometimes postponed due to delayed care seeking for alarm symptoms, regularly caused by unresolved language barriers, as one midwife described:

*“I once experienced a situation where a woman had a lot of blood loss*,* but she only spoke – well*,* I don’t know which language*,* but let’s say Tigrinya*,* and there was no one around her at that moment who could speak English. So it took about two hours until someone was able to call me on her behalf. By the time I arrived*,* it was already too late because there was indeed something wrong with the baby*,* causing her excessive bleeding. But because she simply didn’t know how to reach us due to a language barrier*,* she just didn’t call. And things went wrong.”* (Midwife 11).

Midwives tried to improve timely access to care by organizing consultation hours at or near asylum reception centers, providing antenatal care in a group format, and actively contacting women after missed appointments, at times in collaboration with COA and GZA.

#### Interdisciplinary collaboration

Barriers to interdisciplinary collaboration in pregnancy care for asylum seekers commonly included difficult communication with COA and GZA, as well as limited dissemination and implementation of the national guidelines in all involved organizations. In addition, several midwives voiced concerns regarding the inconsistent quality of care when they referred clients to care providers who had less experience with forcibly displaced women. Specifically, multiple midwives observed a lack of time and compassion for these clients during consultations in the hospital:

“*Actually*,* it sounds very rough*,* but a part of the humane – is lacking there [in the hospital]*,* and that truly breaks my heart*,* when someone just says afterwards: ‘but that was no discussed with me at all*,* I didn’t want that*,* and I had put it in a plan with you.’ It just breaks me when I see that*,* and the communication as well – not much effort is made to guide them [the clients] in a proper and correct way. And that puts them – that is a very vulnerable position as a birthing woman.”* (Midwife 5).

In regions where the national guideline for care for asylum seekers was more widely implemented, midwives highlighted its benefits for interdisiciplinary collaboration, citing specific aspects such as regular meetings with all parties involved in care. Implementation of was mostly achieved through the development of local protocols, based on the national guideline:

*“We initially ran into the issue that everything was just poorly organized. And then I thought: ‘I can either sit and complain*,* or I can just say: hey*,* hello*,* I’m (first name)*,* shall we create a protocol together with the AZC [asylum reception center]. (…) And at that time*,* a new [national] guideline had just come out on care for asylum seekers*,* and we turned that into a protocol. We then presented it to the collaborative network with the hospitals*,* and they approved it*,* and now it’s in place at the AZC*,* yes*,* with the COA.”* (Midwife 6).

In addition, several midwives indicated the necessity of having designated contact persons and dedicated staff at COA, GZA, the hospital, and postpartum care organizations to facilitate efficient interdisciplinary collaboration.

### 3. Barriers in the context of forcibly displaced women

The third level of barriers included the broader context that affected the lived realities of forcibly displaced women under midwives’ care. Specifically, these barriers were related to the housing conditions, the resettlement process, and the prevalent mental health issues of this population.

#### Housing conditions

Most midwives were concerned about the housing conditions in reception centers for asylum seekers, which they deemed unsuitable for pregnant women in terms of hygiene, food options, space, and privacy. The majority of midwives described feelings of compassion and powerlessness in relation to women’s living conditions, frequent relocations, and the stress of asylum procedures:

“*If you see someone saying that she feels depressed because of the burden from COA*,* that she may be deported*,* that she will be relocated to another center and that she feels unsafe*,* that she cannot return to her country of origin because she would be threatened with death. When you witness that happening right in front of you*,* you think*,* ‘Oh my God*,* that woman truly cannot go back because she will simply be killed*. (…) *Yeah*,* it truly moves you’*” (Midwife 3).

Several midwives expressed their efforts to advocate for clients’ needs, such as requesting improved living conditions from COA or writing letters to clients’ lawyers. Others considered such efforts to fall beyond their professional responsibilities. For women with a residence permit, midwives often requested hospital birthing beds via ‘social indication’ to prevent suboptimal homebirths.

#### Resettlement after forced migration

Many midwives encountered barriers in supporting clients in the process of resettlement after forced migration. These included the lack of social support networks, unfamiliarity with the healthcare system and other institutions, and clients’ vulnerable socioeconomic position. Midwives also highlighted the lack of adequate guidance for women who recently transitioned out of the asylum system with a residence permit:

“*As soon as they [forcibly displaced women] leave the asylum center*,* they are suddenly assumed to be normal people. They are still the same individuals who don’t understand certain things*,* and there are still things that need to be arranged.”* (Midwife 6).

To tailor care to women in the process of resettlement, midwives organized practical, material, and social support for their clients. This included collecting donations from former clients or coordinating informal peer support within their practice. Two midwives also implemented formal antenatal group care for forcibly displaced clients.

#### Mental health

The majority of midwives highlighted the gap between the mental health needs of forcibly displaced clients and the services available. In the Netherlands, the waiting periods for psychological treatments are generally lengthy, and while hospitals provide specialized perinatal mental healthcare, midwives reported barriers in access for forcibly displaced women due to language barriers or the physical distance of the hospital. Midwives described that women would regularly share “*horrifying”* experiences (midwife 10), while most midwives felt unprepared to offer appropriate psychological support:

*“Of course*,* that is not our profession. And it’s also too intense*,* you know*,* sometimes the tears are almost in your eyes when you hear what they have been through.”* (Midwife 2).

Nevertheless, many midwives outlined their strategies and dedication to positively influencing women’s mental well-being and pregnancy experience, mostly by fostering a safe and supportive environment:

*“Because I feel they are vulnerable and have been through so much*,* leaving so much behind*,* that I think: I truly want this to be place of comfort for you*,* where you feel safe. (…) Because then*,* I think they can give birth in a good way as well*,* and the child can have a good start.”* (Midwife 2).

### Core theme: imbalance between midwives’ responsibilities and resources

The numerous barriers across different levels of care for forcibly displaced women ultimately culminated in a core theme of imbalance between midwives’ responsibilities and resources. Almost all midwives described taking on additional responsibilities and workloads to navigate barriers, often extending beyond their typical role in “*regular care*” (midwife 7):

*“[When caring for forcibly displaced women] you feel like a social worker*,* you feel like a planner*,* you feel like someone’s buddy*,* you feel like a psychologist – you just have a lot more roles than only your profession.”* (Midwife 3).

Juggling roles, midwives stretched their boundaries to meet their clients’ needs in different ways, as one mentioned, “*not always strictly following the rules”* (midwife 1). The impression of forcibly displaced clients as “*drowning in an enormous system*” (midwife 1), “*disoriented*,” (midwife 3) or “*not capable enough [to be responsible for organizing their own care]*” (midwife 2) strengthened midwives’ sense of duty to adopt an increased level of responsibility.

However, midwives simultaneously reported a shortage of the resources required to apply supportive strategies for meeting their clients’ needs. These resources included extra time, financial compensation and supportive services, as well as more intangible aspects such as power and influence, related to their ability to advocate for their clients and navigate the healthcare system on their behalf. The resulting imbalance was frequently experienced as an increased emotional burden:

*“You only experience that sense of peace and satisfaction when someone has given birth*,* and you see them safe*,* with a child*,* after a good birth. (…) Only then can you really let go of the pressure of providing support. As long as someone is still pregnant*,* and you have to manage a lot of things*,* and you get stressed when someone calls with complaints that are related to stress but you can’t do anything about it – it really does provide extra satisfaction when it’s all over. But as long as you’re busy*,* it can be quite a burden.” (*Midwife 4).

## Discussion

This qualitative study sheds light on the experiences of midwives caring for forcibly displaced women in the Netherlands. From these experiences, we identified barriers to equitable care at the levels of midwife-client interactions, the organization of care, and the wider context of forcibly displaced women. As a result, midwives were burdened with additional responsibilities and roles, while they had limited resources and capacity to fully respond to women’s needs. Our results provide insights into the underlying dynamics and potential solutions to address this imbalance, and ultimately enhance midwives’ influence on the wellbeing of forcibly displaced women in pregnancy and childbirth care.

The barriers identified in this study echo previous research in different settings of care for migrant populations [17–20]. In particular, our findings add to the evidence of the detrimental impact of issues such as unresolved language barriers and frequent relocations of asylum seekers on care quality [21–23]. As observed in other studies, midwives developed different strategies and frequently adopted more responsibilities in reaction to these multi-level barriers [18]. 

However, our results also confirmed that these strategies did not always result in optimal, equitable care. For instance, barriers regarding interpreter services left midwives relying on communication strategies that compromised legal and clinical standards. Through such practices, care may inadvertently further marginalize forcibly displaced women by silencing their voices and endangering maternal safety [24, 25]. In our study, midwives also inadvertently engaged in cultural stereotyping, in line with previous studies in other countries [17, 26, 27]. Among stereotypical notions, midwives’ perception of forcibly displaced women as easily grateful clients may mask social desirability or lowered expectations of care among this population [28]. This illustrates how implicit bias or cultural “othering” may lead midwives to misinterpret or generalize the diverse needs of forcibly displaced women [29]. Consequently, this can discourage women from seeking care and lead to incomplete provision of information [26, 29]. 

Most barriers that midwives encountered in our study are deeply rooted in the broader social, political, and economic structures that shape the circumstances of forcibly displaced pregnant women. These structures include current migration policies, while entangled with historical legacies of colonization, xenophobia and gendered racism, which continue to drive forced displacement today. Thereby, our study elucidated a critical dynamic in care: while midwives were often overwhelmed by immediate client needs, they often fight an uphill battle against systemic barriers that impede equitable care. For instance, midwives’ efforts to create a safe and trusting environment were undermined by the stress and uncertainty caused by lengthy asylum procedures. In other cases, midwives resorted to using their own car to transport women to the hospital, while persistent issues with the taxi services contracted at the asylum reception centers remained unresolved.

Pushed toward temporary solutions and assuming additional roles, midwives may face an increased risk of demoralization and burnout [30]. To address the imbalance in current midwifery care for forcibly displaced women, midwives therefore need to be supported by additional resources, such as funding for doulas or antenatal group care. However, midwives must also gain the knowledge, tools and critical consciousness to recognize and address the structural inequities that affect the women they care for, but also place a heavy burden on midwives themselves. Recently, the field of “Critical Midwifery Studies” was launched to promote concepts such as reproductive justice in midwifery education and practice, empowering midwives to offer equitable care and advocate for structural changes [31]. 

The specific levels of barriers we identified provide several directions for supporting midwives in providing more equitable care. On the interactional level, the reimplementation of state-funded professional interpreter services in the Netherlands, which was realized after this study following their initial abolition, represents a crucial step forwards. However, our results emphasize that enhancing the quality and availability of these services, along with training midwives’ to work with interpreters and offer culturally sensitive care, remains necessary [32]. In addition, the introduction of cultural mediators or peer support programs could help to bridge communication barriers beyond language. Unfortunately, recent Dutch research highlighted significant admission biases in midwifery education, disadvantaging applicants with migration backgrounds [33]. Efforts to promote a more representative professional workforce and facilitate cultural concordance in care are thus essential [34]. 

On the organizational level of care, policy reforms should urgently minimize relocations of pregnant women within asylum reception centers, given how issues such as unstable housing and transportation affect women’s wellbeing and the quality of midwifery care. Wider implementation of the existing guideline for women in asylum reception centers can help boost collaboration and quality across all care disciplines involved. In addition, further professional standards for care for women transitioning out of the asylum system is imperative as standardized practices are now largely absent for this group. To improve care organization, additional barriers that may explain suboptimal care, such as frequently changing contracts for primary care in asylum reception centers and the geographical isolation of these centers, should also be explored and addressed.

On the contextual level, our study implies that improving the living conditions of women in asylum reception centers is a prerequisite for meeting basic health and safety needs [35, 36]. In addition, the socioeconomic vulnerability that often accompanies forced migration could be structurally improved through quicker access to formal language education, labor participation, regular housing and naturalization [37, 38]. Previous research showed how positive integration policies can improve the birth outcomes of different migrant populations, as well as their mental health [39]. Regarding the substantial unmet mental health needs among forcibly displaced women that midwives in this study encountered, access to mental health screening and services are priorities [40, 41]. Although midwives cannot replace specialized mental healthcare providers, our findings indicate that principles of trauma-informed care can be implemented, concentrating on women’s emotional safety. Specific training in such approaches could help reduce the risk of retraumatization and make the midwifery practice a safer space forcibly displaced women [42]. 

### Strengths and limitations

To the best of our knowledge, this was the first qualitative study that centered the experiences of Dutch community midwives with two specific populations of forcibly displaced women. We included a diverse sample of midwives from various Dutch regions and with different degrees of working experience with women in asylum reception centers and those with a refugee residence permit. However, our findings may still reflect a limited range of perspectives, assuming that midwifery practices that agreed to participate in the EGALITE project are specifically motivated to improve care for forcibly displaced populations. Furthermore, future research should aim to include a wider array of voices, particularly those of forcibly displaced women themselves, to enrich the understanding of priorities for care improvement [34, 43]. 

## Conclusions

This qualitative study shed light on the barriers in midwifery care for forcibly displaced women in the Netherlands, revealing a critical imbalance between responsibilities shouldered by midwives and the resources available to tailor care to women’s needs. To address this imbalance, it is crucial to dismantle barriers ranging from the individual encounters in the consultation room to the circumstances of forcibly displaced women during their pregnancy and childbirth. Specific policy priorities should include enhancing the quality and availability of state-funded interpreter services, improving women’s living conditions during resettlement, and facilitating access to support such as specialized mental health services. It is also imperative to confront and mitigate the structural determinants that contribute to the imbalance in midwives’ work, such as migration and socioeconomic policies that marginalize forcibly displaced women and restrict the scope of midwives’ influence on their well-being. Incorporating self-reflective practices and concepts of reproductive justice into midwifery education and practice will be essential to equip midwives to engage more critically with the systemic barriers they face, and advocate for structural change. Ultimately, only through a comprehensive understanding of and engagement with the root causes can we hope to break the cycle of inequity and ensure that midwifery care is equally safe for everyone.

## Electronic supplementary material

Below is the link to the electronic supplementary material.

Supplementary Material 1.

Supplementary Material 2.

## Data Availability

The data supporting this study are available upon request, subject to certain conditions. Requests for access can be considered for researchers with similar objectives to those of this study.

## Abbreviations

AZC: Asylum reception center
COA: Dutch Central Agency for the Reception of Asylum Seekers
GZA: GZA Healthcare (provider currently contracted by COA for primary healthcare at most asylum reception centers)
