# Supplementary material for: Let this be a safe place: a qualitative study into midwifery care for forcibly displaced women in the Netherlands
Source: BMC Health Serv Res. 2024 Nov 29;24:1503. doi: 10.1186/s12913-024-11852-w (PMC11605984; doi:10.1186/s12913-024-11852-w)
Supplement: Supplementary file 1 — Supplementary Material 1. [file 12913_2024_11852_MOESM1_ESM.docx]

# **Let this be a safe place: a qualitative study into midwifery care for forcibly displaced women in the Netherlands**

# Appendix 2: Code tree

*Theme 1: midwife-client interactions (micro-level)*

| **Subtheme** | **What barriers do midwives encounter in care?** | **How do midwives manage/adjust care to overcome these barriers?** | |
| --- | --- | --- | --- |
| Language and interpretation | | - Limited access to information - Limited expression and (fear of) misunderstandings - Difficult counseling during birth - Empathy/deep connections more difficult with interpreters - Risk of miscommunications with formal and informal interpreters - Limited quality and availability of professional interpreter services - No funding for interpreter services for recognized refugees | - Working with professional interpreters as much as possible - Working with female interpreters - Limiting professional services to essential moments in care (or reserving extra time) - Accepting alternatives to interpreters: body language, multilanguage materials, buddies, speaking different languages |
| Cultural differences | | - FDP have different expectations of care than Dutch clients - FDP do not express their needs and questions as openly as Dutch clients - Midwives’ limited competences/cultural stereotyping | - Positive dialogue/learning from differences - Feeling motivated by grateful clients - Emphasizing similarities instead of differences |
| Building trust | | - Building trust takes more time - FDP more fearful than other clients (e.g. fear of immigration authorities) - Lack of continuity of care | - Continuity of care - Showing patience and genuine interest in client - Offering safe atmosphere - Shared religious background |

*Theme 2: organization of care (meso-level)*

| **Subtheme** | **What barriers do midwives encounter in care?** | **How do midwives manage/adjust care to overcome these barriers?** | |
| --- | --- | --- | --- |
| Delays in care-seeking and access to care | - Late start of care with incomplete patient records - High frequency of missed appointments - Delayed contact for emergency symptoms (e.g. due to language barriers) - Lack of transportation options and unreliable taxi services - Relocations of asylum seekers | - Individual or group antenatal care at the reception center - Active follow-up after missed appointments - Close contact with other professionals (e.g. GZA) - Using personal vehicle to transport clients |  |
| Relocations of asylum seekers | - Lack of communication by COA - Incomplete or untimely transfer of information - Losing clients/lack of overview - Physical burden pregnant women - Rule of 34weeks not always adhered to | - No relocations between 34w – 7w postpartum - Requesting a relocation blockade for medical reasons - Ensuring handover of relocated clients to subsequent care provider | |
| Interdisciplinary collaboration | - Concerns about quality of care, especially in the hospital - Difficult communication with COA and/or GZA - Limited guideline awareness and implementation | - Implementing guideline (e.g. multidisciplinary meetings) - Short lines of communication (e.g. contact person at each organization) - Committed colleagues at other midwifery practices/GZA/postpartum care providers/hospital | |

*Theme 3: living conditions of forcibly displaced women*

| **Subtheme** | **What barriers do midwives encounter in care?** | **How do midwives manage/adjust care to overcome these barriers?** |
| --- | --- | --- |
| Housing conditions | - Poor hygiene, limited food options, lack of privacy, and other conditions unsuitable for pregnant women in asylum reception centers - Stress of asylum procedures and threat of deportation | - Advocating for better living circumstances in asylum centers - Arranging hospital births |
| Resettlement after forced migration | - Social isolation - Unfamiliarity with healthcare system - Lack of guidance for refugees with permit - Vulnerable socioeconomic position | - Involving doulas or buddy projects - Providing group care and stimulating peer support - Organizing practical and material support |
| Mental health | - High burden of trauma-related symptoms and other mental health conditions - Specialized mental healthcare not available/accessible for refugees | - Creating safety for women to discuss mental health |

**Central theme: imbalance in midwives’ work**

Subthemes:

- Perceived need to take over responsibility and transcend usual role
- Increased practical and emotional burden
- Limited influence on clients’ health and wellbeing
- Limited resources, e.g. time, power, financial compensation, skills/education, supportive services, referral options
