# Supplementary material for: Let this be a safe place: a qualitative study into midwifery care for forcibly displaced women in the Netherlands
Source: BMC Health Serv Res. 2024 Nov 29;24:1503. doi: 10.1186/s12913-024-11852-w (PMC11605984; doi:10.1186/s12913-024-11852-w)
Supplement: Supplementary file 2 — Supplementary Material 2. [file 12913_2024_11852_MOESM2_ESM.docx]

# **Let this be a safe place: a qualitative study into midwifery care for forcibly displaced women in the Netherlands**

# Appendix 1: Field visit description and topic guide (final English language version)

**Description of the field visits, conducted as part of developing the topic guide.**

*The field visits were conducted by the first author [JT] and a research student. One visit involved a day of consultations at an asylum reception center by a local midwifery practice. Two visits took place at a midwifery practice, where asylum seekers and recognized refugees attended regular consultation hours. A fourth visit was to an asylum reception center, where the researchers met with a doula working there and a midwife from a nearby practice. During these visits, the researchers engaged in informal conversations and observed midwives in their daily interactions with forcibly displaced clients. In addition to topics taken from the literature, several relevant areas emerged from these field observations, which were then incorporated into the topic guide. For example, some midwives routinely inquired about women's migration history, while others did not. Exploration of this variation was subsequently included with the example questions in the topic guide.*

| **Category** | **Topic** | **Example questions (including possible probes, applied flexibly and depending on question response)** |
| --- | --- | --- |
| Introduction | Introduction researchers  Informed consent procedure  Check completion of midwife’s information sheet |  |
| Organization of care | General (regional) organization of care  Collaboration/coordination  Specific organizational aspects | - How did you/your midwifery practice become involved with care for asylum seekers and refugees with residence permit? - How is care for asylum seekers/refugees organized in your practice and in the region? (What are roles and responsibilities? How would you describe the collaboration with other care providers?) - Could you tell me more about specific organizational aspects (e.g., the use of guidelines, collaboration with the hospital or asylum reception center)? |
| Experiences | General and personal experiences with care for asylum seekers and refugees  Workload | - Could you describe your experiences with providing care to asylum seekers/refugees? (How do you personally feel about working with asylum seekers and/or refugees?) - How do you perceive the workload of care for this population? (What makes it that way (e.g., demanding/rewarding)?) |
| Tailored care | Comparing and adjusting care  Relevant differences  Health disparities | - How would you compare caring for asylum seekers to caring for refugees with a residence permit? And to what extent/in what ways do these populations have different needs than other women in your practice? (What are differences or similarities? What characteristics of women determine different needs among and between groups? How do you adjust your care based on these differences?) - Do you ask women about their migration backgrounds/histories? (Can you give examples of how women’s migration backgrounds influence the way you provide care?) - What barriers do you think newly arrived women experience on their way through the Dutch perinatal care system? (How can midwives support them best? To what extent do you feel responsible?) - What do you think of the pregnancy outcomes of women with a refugee background? (What possible explanations do you see for differences between them and Dutch women? Would you say refugee women have higher risks/are vulnerable and why (not)?) |
| Challenges in care | Quality of care  General challenges and specific barriers  Views on previously identified challenges | - How do you view the quality of care to this group, compared to other groups? (In case of suboptimal care, what might be the causes? What best practices or suboptimal care factors do you perceive?) - What challenges do you experience in care for asylum seekers/refugees? (What are the most important barriers? What effects do these barriers have?) - How do you view challenges we previously identified in care (e.g., in communication with clients, interdisciplinary collaboration, psychosocial care, and the vulnerable situation of asylum seekers/refugees in the Netherlands)? |
| Strategies and best practices | Dealing with challenges  Example best practices or solutions  Improvements in care | - How do you deal with these challenges? (What support or solutions do you have (or miss)?) - Do you have positive examples, or recommendations to other midwives? - Do you see potential improvements that could be made? (If you could change something tomorrow, what would be the first thing?) - What do you think should be different in the care for asylum seekers/refugees in five years? What should we maintain? (How do you think midwives can best be supported/what do midwives need in the future?) - What are your views on possible improvements (e.g., availability of interpreter services, training for midwives, introducing interventions such as group antenatal care or buddy programs)? |
| Completion | Summarizing/following-up on unclarities, unaddressed field visit observations  Check if interviewee would like to receive transcript  Thanking interviewee | - In the field visit to your practice, we noticed that (…), what is your view on (…) ? - Do you feel that we have gained an understanding of your experience with and ideas about the care for asylum seekers and refugees in this interview? - Are there any important matters that have not been addressed? (Do you want to add anything? What would you like us to focus on in the research?) |
